# *N*′-(2-Hy­droxy-3,5-diiodo­benzyl­idene)-2-methyl­benzohydrazide

**DOI:** 10.1107/S1600536810052050

**Published:** 2010-12-18

**Authors:** Chun-Bao Tang

**Affiliations:** a Department of Chemistry, Jiaying University, Meizhou 514015, People’s Republic of China

## Abstract

The asymmetric unit of the title compound, C_15_H_12_I_2_N_2_O_2_, contains two independent mol­ecules in which the dihedral angles between the two benzene rings are 62.4 (7) and 41.1 (7)°. Intra­molecular O—H⋯N hydrogen bonds generate *S*(6) ring motifs in each mol­ecule. In the crystal, mol­ecules are linked through inter­molecular N—H⋯O hydrogen bonds, forming chains along the *a* axis.

## Related literature

For general background to hydrazones, see: Rasras *et al.* (2010 ▶); Pyta *et al.* (2010 ▶); Angelusiu *et al.* (2010 ▶). For related structures, see: Fun *et al.* (2008 ▶); Singh & Singh (2010 ▶); Ahmad *et al.* (2010 ▶); Tang (2010 ▶). For reference bond-length data, see: Allen *et al.* (1987 ▶) and for hydrogen-bond motifs, see: Bernstein *et al.* (1995 ▶).
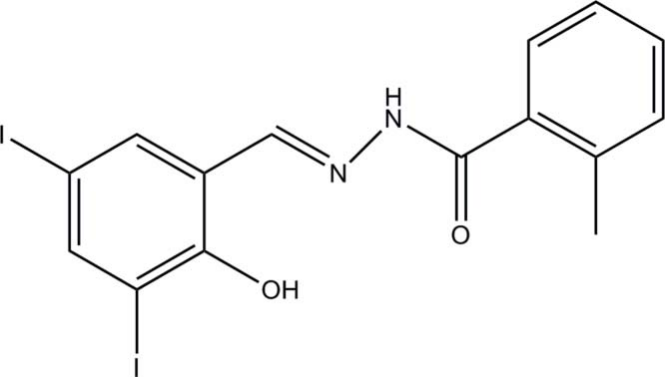


## Experimental

### 

#### Crystal data

C_15_H_12_I_2_N_2_O_2_
*M* *_r_* = 506.07
Monoclinic, 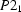

*a* = 9.658 (2) Å
*b* = 11.723 (2) Å
*c* = 14.732 (3) Å
β = 93.216 (2)°
*V* = 1665.4 (6) Å^3^
*Z* = 4
Mo *K*α radiation
μ = 3.78 mm^−1^
*T* = 298 K
0.18 × 0.17 × 0.15 mm

#### Data collection

Bruker SMART CCD area-detector diffractometer
Absorption correction: multi-scan (*SADABS*; Sheldrick, 1996 ▶) *T*_min_ = 0.549, *T*_max_ = 0.601
12106 measured reflections
5976 independent reflections
4443 reflections with *I* > 2σ(*I*)
*R*_int_ = 0.040

#### Refinement

*R*[*F* ^2^ > 2σ(*F* ^2^)] = 0.043
*wR*(*F* ^2^) = 0.089
*S* = 1.01
5976 reflections
389 parameters
3 restraints
H atoms treated by a mixture of independent and constrained refinement
Δρ_max_ = 0.53 e Å^−3^
Δρ_min_ = −0.88 e Å^−3^
Absolute structure: Flack (1983 ▶), 2163 Friedel pairs
Flack parameter: 0.10 (3)

### 

Data collection: *SMART* (Bruker, 2002 ▶); cell refinement: *SAINT* (Bruker, 2002 ▶); data reduction: *SAINT*; program(s) used to solve structure: *SHELXS97* (Sheldrick, 2008 ▶); program(s) used to refine structure: *SHELXL97* (Sheldrick, 2008 ▶); molecular graphics: *SHELXTL* (Sheldrick, 2008 ▶); software used to prepare material for publication: *SHELXL97*.

## Supplementary Material

Crystal structure: contains datablocks global, I. DOI: 10.1107/S1600536810052050/sj5075sup1.cif

Structure factors: contains datablocks I. DOI: 10.1107/S1600536810052050/sj5075Isup2.hkl

Additional supplementary materials:  crystallographic information; 3D view; checkCIF report

## Figures and Tables

**Table 1:** Hydrogen-bond geometry (Å, °)

*D*—H⋯*A*	*D*—H	H⋯*A*	*D*⋯*A*	*D*—H⋯*A*
O1—H1⋯N1	0.82	1.87	2.570 (9)	143
O3—H3⋯N3	0.82	1.85	2.560 (10)	144
N4—H4⋯O2	0.90 (5)	1.94 (4)	2.786 (8)	155 (8)
N2—H2⋯O4^i^	0.90 (6)	1.91 (3)	2.788 (9)	164 (10)

Symmetry code: (i) 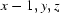.

**Fig. 1.**
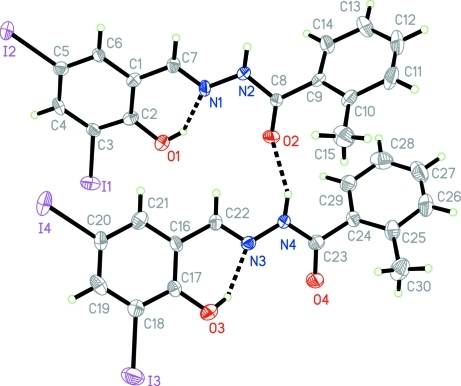
The molecular structure of the compound, showing the atom-numbering scheme. Displacement ellipsoids are drawn at the 30% probability level. Hydrogen atoms are shown as spheres of arbitrary radius and hydrogen bonds are drawn as dashed lines.

**Fig. 2.**
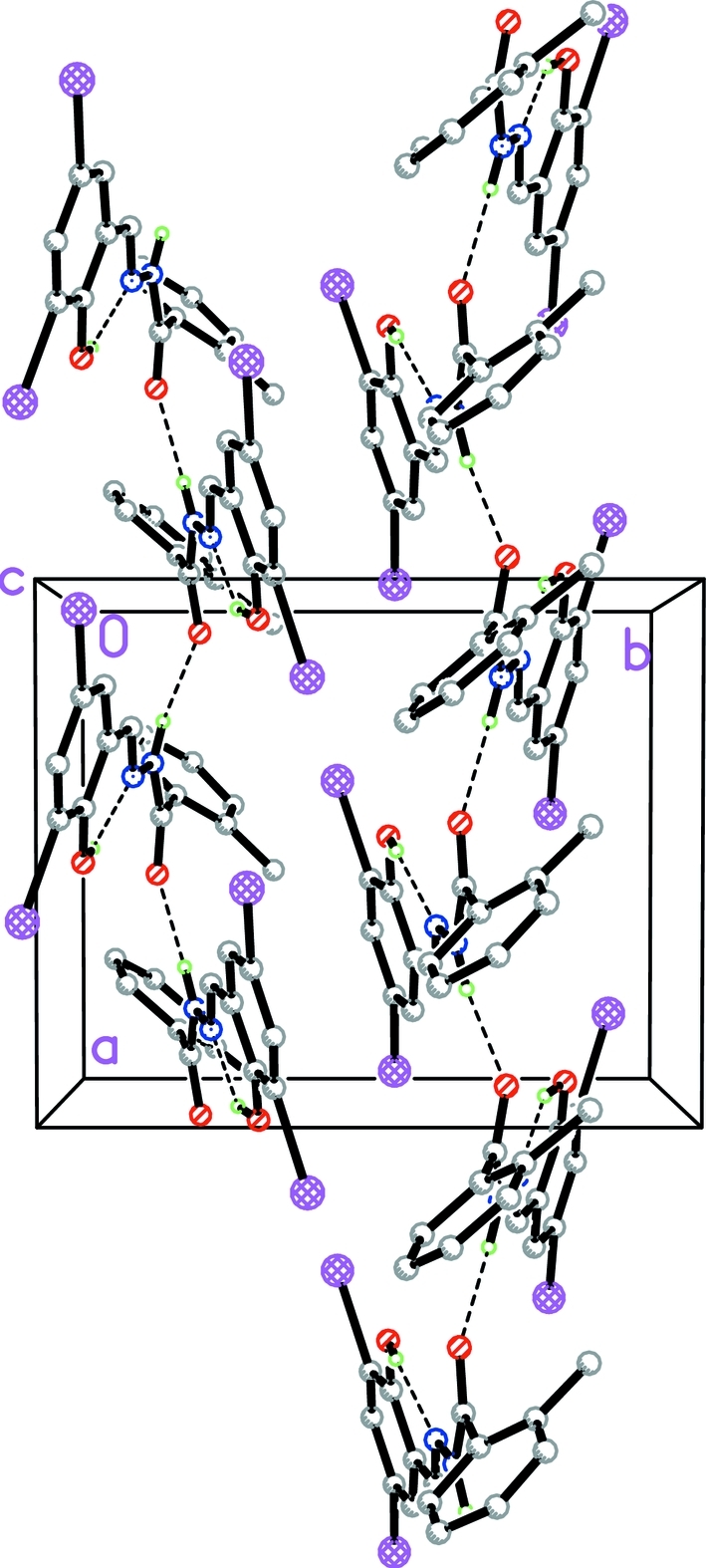
Molecular packing of the title compound, with hydrogen bonds shown as dashed lines.

## supplementary crystallographic information

### Comment

Hydrazone compounds have received much attention in biological and structural chemistry in the last few years (Rasras *et al.*, 2010; Pyta *et al.*, 2010; Angelusiu *et al.*, 2010; Fun *et al.*, 2008; Singh & Singh, 2010; Ahmad *et al.*, 2010). In the present paper, the author reports the crystal structure of the new title hydrazone compound (Fig. 1).

The asymmetric unit of the title compound contains two independent molecules. The dihedral angles between the two benzene rings in the two molecules are 62.4 (7) and 41.1 (7)°, respectively. The torsion angles C1—C7—N1—N2, C7—N1—N2—C8, N1—N2—C8—C9, C16—C22—N3—N4, C22—N3—N4—C23, and N3—N4—C23—C24 are 1.5 (6), 7.2 (6), 4.9 (6), 3.4 (6), 4.8 (6), and 4.3 (6)°, respectively. Bond lengths in the molecules are normal (Allen *et al.*, 1987) and comparable to those in the similar compound the author reported recently (Tang, 2010). Intramolecular O1—H1···N1 and O3—H3···N3 hydrogen bonds generate S(6) ring motifs in each molecule (Bernstein *et al.*, 1995). In the crystal structure, molecules are linked through intermolecular N—H···O hydrogen bonds (Table 1), forming chains along the *a* axis (Fig. 2).

### Experimental

2-Hydroxy-3,5-diiodobenzaldehyde (0.1 mmol, 37.4 mg) and 2-methylbenzohydrazide (0.1 mmol, 15.0 mg) were dissolved in methanol (20 ml). The mixture was stirred at reflux for 10 min to give a clear colourless solution. Colourless block-shaped crystals of the compound were formed by slow evaporation of the solvent over several days.

### Refinement

The amino H atoms were located in a difference Fourier map and refined isotropically, with the N—H distances restrained to 0.90 (1) Å [*U*_iso_(H) = 0.08 Å^2^]. Other H atoms were constrained to ideal geometries and refined as riding, with Csp^2^—H = 0.93 Å, C(methyl)—H = 0.96 Å, and O—H = 0.82 Å; *U*_iso_(H) = 1.2*U*_eq_(C) and 1.5*U*_eq_(O and C_methyl_).

### Crystal data

**Table:**

C_15_H_12_I_2_N_2_O_2_	*F*(000) = 952
*M**_r_* = 506.07	*D*_x_ = 2.018 Mg m^−^^3^
Monoclinic, *P*2_1_	Mo *K*α radiation, λ = 0.71073 Å
Hall symbol: P 2yb	Cell parameters from 3095 reflections
*a* = 9.658 (2) Å	θ = 2.5–24.5°
*b* = 11.723 (2) Å	µ = 3.78 mm^−^^1^
*c* = 14.732 (3) Å	*T* = 298 K
β = 93.216 (2)°	Block, colourless
*V* = 1665.4 (6) Å^3^	0.18 × 0.17 × 0.15 mm
*Z* = 4	

### Data collection

**Table:**

Bruker SMART CCD area-detector diffractometer	5976 independent reflections
Radiation source: fine-focus sealed tube	4443 reflections with *I* > 2σ(*I*)
graphite	*R*_int_ = 0.040
ω scans	θ_max_ = 27.0°, θ_min_ = 2.1°
Absorption correction: multi-scan (*SADABS*; Sheldrick, 1996)	*h* = −12→12
*T*_min_ = 0.549, *T*_max_ = 0.601	*k* = −9→14
12106 measured reflections	*l* = −18→18

### Refinement

**Table:**

Refinement on *F*^2^	Secondary atom site location: difference Fourier map
Least-squares matrix: full	Hydrogen site location: inferred from neighbouring sites
*R*[*F*^2^ > 2σ(*F*^2^)] = 0.043	H atoms treated by a mixture of independent and constrained refinement
*wR*(*F*^2^) = 0.089	*w* = 1/[σ^2^(*F*_o_^2^) + (0.0325*P*)^2^] where *P* = (*F*_o_^2^ + 2*F*_c_^2^)/3
*S* = 1.01	(Δ/σ)_max_ < 0.001
5976 reflections	Δρ_max_ = 0.53 e Å^−^^3^
389 parameters	Δρ_min_ = −0.88 e Å^−^^3^
3 restraints	Absolute structure: Flack (1983), 2163 Friedel pairs
Primary atom site location: structure-invariant direct methods	Flack parameter: 0.10 (3)

### Special details

**Table:**

Geometry. All esds (except the esd in the dihedral angle between two l.s. planes) are estimated using the full covariance matrix. The cell esds are taken into account individually in the estimation of esds in distances, angles and torsion angles; correlations between esds in cell parameters are only used when they are defined by crystal symmetry. An approximate (isotropic) treatment of cell esds is used for estimating esds involving l.s. planes.
Refinement. Refinement of F^2^ against ALL reflections. The weighted R-factor wR and goodness of fit S are based on F^2^, conventional R-factors R are based on F, with F set to zero for negative F^2^. The threshold expression of F^2^ > 2sigma(F^2^) is used only for calculating R-factors(gt) etc. and is not relevant to the choice of reflections for refinement. R-factors based on F^2^ are statistically about twice as large as those based on F, and R- factors based on ALL data will be even larger.

### Fractional atomic coordinates and isotropic or equivalent isotropic displacement parameters (Å^2^)

**Table:**

	*x*	*y*	*z*	*U*_iso_*/*U*_eq_	
I1	0.63728 (6)	−0.05063 (8)	0.65864 (5)	0.0703 (3)	
I2	0.04006 (6)	0.04601 (6)	0.72932 (4)	0.0560 (2)	
I3	1.16416 (8)	0.40275 (8)	0.64944 (6)	0.0777 (3)	
I4	0.57051 (8)	0.30877 (7)	0.73109 (5)	0.0722 (3)	
N1	0.3538 (7)	0.1082 (8)	0.3438 (4)	0.040 (2)	
N2	0.3260 (6)	0.1360 (8)	0.2538 (5)	0.039 (2)	
N3	0.8691 (7)	0.2375 (8)	0.3398 (5)	0.037 (2)	
N4	0.8323 (6)	0.2070 (7)	0.2514 (5)	0.038 (2)	
O1	0.5262 (5)	0.0329 (7)	0.4690 (4)	0.0500 (18)	
H1	0.4996	0.0449	0.4160	0.075*	
O2	0.5548 (5)	0.1423 (9)	0.2297 (4)	0.065 (3)	
O3	1.0418 (5)	0.3199 (8)	0.4607 (4)	0.055 (2)	
H3	1.0188	0.2846	0.4142	0.082*	
O4	1.0562 (5)	0.2148 (8)	0.2173 (4)	0.062 (3)	
C1	0.2840 (8)	0.0766 (8)	0.4902 (6)	0.035 (2)	
C2	0.4176 (7)	0.0386 (10)	0.5221 (6)	0.038 (2)	
C3	0.4385 (7)	0.0046 (8)	0.6126 (6)	0.038 (2)	
C4	0.3328 (8)	0.0075 (8)	0.6722 (5)	0.041 (3)	
H4A	0.3488	−0.0147	0.7325	0.050*	
C5	0.2011 (7)	0.0447 (10)	0.6398 (6)	0.041 (2)	
C6	0.1778 (8)	0.0739 (8)	0.5498 (5)	0.036 (2)	
H6	0.0883	0.0925	0.5283	0.044*	
C7	0.2561 (8)	0.1083 (9)	0.3969 (6)	0.037 (2)	
H7	0.1670	0.1288	0.3759	0.044*	
C8	0.4346 (7)	0.1479 (9)	0.1991 (5)	0.034 (2)	
C9	0.3917 (8)	0.1684 (10)	0.1014 (6)	0.043 (3)	
C10	0.4529 (9)	0.2538 (8)	0.0538 (6)	0.048 (2)	
C11	0.4131 (11)	0.2638 (10)	−0.0406 (6)	0.070 (3)	
H11	0.4512	0.3210	−0.0751	0.084*	
C12	0.3196 (13)	0.1896 (14)	−0.0802 (8)	0.086 (5)	
H12	0.2945	0.1972	−0.1417	0.103*	
C13	0.2618 (11)	0.1041 (11)	−0.0316 (7)	0.079 (3)	
H13	0.1987	0.0541	−0.0604	0.095*	
C14	0.2961 (9)	0.0924 (9)	0.0577 (6)	0.058 (3)	
H14	0.2568	0.0343	0.0906	0.070*	
C15	0.5496 (10)	0.3374 (10)	0.0925 (7)	0.075 (3)	
H15A	0.5197	0.3620	0.1504	0.113*	
H15B	0.5531	0.4018	0.0524	0.113*	
H15C	0.6402	0.3039	0.1004	0.113*	
C16	0.8046 (8)	0.2772 (9)	0.4902 (5)	0.041 (3)	
C17	0.9381 (8)	0.3161 (10)	0.5183 (5)	0.037 (2)	
C18	0.9635 (8)	0.3486 (9)	0.6068 (6)	0.045 (3)	
C19	0.8592 (10)	0.3491 (9)	0.6676 (6)	0.052 (3)	
H19	0.8777	0.3739	0.7270	0.063*	
C20	0.7290 (9)	0.3129 (10)	0.6401 (6)	0.046 (2)	
C21	0.7013 (9)	0.2749 (9)	0.5519 (6)	0.050 (3)	
H21	0.6135	0.2477	0.5340	0.060*	
C22	0.7737 (9)	0.2397 (10)	0.3971 (6)	0.043 (3)	
H22	0.6841	0.2171	0.3789	0.051*	
C23	0.9330 (8)	0.1963 (10)	0.1929 (6)	0.048 (3)	
C24	0.8872 (8)	0.1720 (9)	0.0985 (6)	0.039 (2)	
C25	0.9316 (9)	0.2342 (8)	0.0260 (6)	0.054 (2)	
C26	0.8796 (12)	0.2072 (11)	−0.0621 (7)	0.068 (3)	
H26	0.9075	0.2497	−0.1112	0.082*	
C27	0.7902 (12)	0.1207 (15)	−0.0763 (8)	0.085 (4)	
H27	0.7588	0.1041	−0.1357	0.102*	
C28	0.7430 (10)	0.0558 (11)	−0.0069 (8)	0.079 (3)	
H28	0.6805	−0.0035	−0.0187	0.094*	
C29	0.7919 (9)	0.0814 (9)	0.0826 (6)	0.057 (3)	
H29	0.7617	0.0391	0.1311	0.069*	
C30	1.0280 (11)	0.3345 (9)	0.0403 (7)	0.076 (3)	
H30A	1.0004	0.3786	0.0911	0.114*	
H30B	1.0239	0.3812	−0.0133	0.114*	
H30C	1.1211	0.3075	0.0522	0.114*	
H2	0.239 (4)	0.153 (10)	0.233 (6)	0.080*	
H4	0.748 (4)	0.188 (10)	0.227 (6)	0.080*	

### Atomic displacement parameters (Å^2^)

**Table:**

	*U*^11^	*U*^22^	*U*^33^	*U*^12^	*U*^13^	*U*^23^
I1	0.0474 (4)	0.0872 (8)	0.0748 (5)	0.0162 (4)	−0.0098 (3)	0.0093 (4)
I2	0.0566 (4)	0.0726 (6)	0.0403 (3)	0.0013 (4)	0.0168 (2)	0.0029 (4)
I3	0.0673 (5)	0.0831 (8)	0.0806 (6)	−0.0164 (4)	−0.0136 (4)	−0.0137 (5)
I4	0.0870 (5)	0.0812 (7)	0.0519 (5)	0.0016 (5)	0.0343 (4)	0.0025 (4)
N1	0.045 (4)	0.051 (7)	0.024 (4)	−0.004 (3)	0.007 (3)	0.004 (3)
N2	0.029 (3)	0.053 (6)	0.035 (4)	0.000 (3)	0.006 (3)	−0.002 (4)
N3	0.034 (4)	0.037 (6)	0.040 (5)	−0.004 (3)	0.002 (3)	0.004 (4)
N4	0.031 (4)	0.054 (6)	0.031 (4)	−0.002 (3)	0.006 (3)	0.001 (4)
O1	0.042 (3)	0.064 (6)	0.045 (4)	−0.001 (3)	0.010 (2)	0.004 (4)
O2	0.026 (3)	0.121 (8)	0.048 (4)	−0.007 (3)	−0.002 (3)	0.007 (4)
O3	0.037 (3)	0.071 (6)	0.058 (4)	−0.002 (4)	0.012 (3)	−0.005 (4)
O4	0.032 (3)	0.101 (8)	0.054 (4)	0.010 (3)	0.004 (3)	−0.010 (4)
C1	0.035 (4)	0.029 (7)	0.042 (5)	−0.004 (4)	0.007 (3)	−0.003 (4)
C2	0.033 (4)	0.031 (7)	0.051 (5)	−0.007 (4)	0.006 (3)	0.000 (5)
C3	0.038 (4)	0.040 (7)	0.036 (5)	0.001 (4)	−0.004 (3)	0.007 (4)
C4	0.062 (5)	0.037 (7)	0.024 (5)	0.001 (4)	−0.005 (4)	0.003 (4)
C5	0.036 (4)	0.042 (7)	0.043 (5)	0.002 (5)	0.002 (3)	0.005 (5)
C6	0.039 (4)	0.042 (8)	0.027 (4)	0.007 (4)	0.000 (3)	0.000 (4)
C7	0.040 (5)	0.036 (7)	0.035 (5)	−0.007 (4)	0.007 (4)	−0.002 (4)
C8	0.030 (4)	0.044 (7)	0.028 (4)	−0.002 (4)	0.003 (3)	−0.001 (4)
C9	0.028 (4)	0.061 (8)	0.040 (5)	0.003 (4)	0.012 (4)	−0.001 (5)
C10	0.044 (5)	0.052 (7)	0.050 (5)	0.009 (4)	0.017 (4)	−0.003 (4)
C11	0.087 (8)	0.080 (9)	0.047 (6)	0.013 (6)	0.035 (5)	0.006 (5)
C12	0.108 (10)	0.118 (15)	0.032 (6)	0.005 (9)	0.014 (6)	−0.004 (7)
C13	0.084 (8)	0.103 (10)	0.048 (6)	−0.004 (7)	−0.015 (5)	−0.020 (6)
C14	0.059 (6)	0.077 (8)	0.037 (5)	−0.007 (5)	−0.001 (4)	−0.010 (5)
C15	0.076 (7)	0.080 (9)	0.070 (7)	−0.014 (6)	0.001 (5)	0.017 (6)
C16	0.044 (5)	0.052 (9)	0.026 (5)	0.003 (4)	−0.001 (3)	−0.001 (4)
C17	0.050 (5)	0.029 (6)	0.032 (5)	0.002 (4)	−0.001 (3)	0.007 (5)
C18	0.044 (5)	0.044 (8)	0.047 (6)	0.009 (4)	0.004 (4)	0.005 (5)
C19	0.081 (7)	0.034 (8)	0.039 (6)	0.001 (5)	−0.014 (5)	−0.002 (5)
C20	0.062 (6)	0.045 (7)	0.031 (5)	−0.002 (5)	0.010 (4)	0.000 (5)
C21	0.050 (5)	0.046 (8)	0.055 (7)	0.006 (5)	0.014 (4)	0.003 (5)
C22	0.047 (5)	0.050 (8)	0.032 (5)	−0.004 (5)	0.006 (4)	−0.001 (5)
C23	0.033 (5)	0.063 (9)	0.049 (6)	0.010 (5)	0.006 (4)	−0.004 (5)
C24	0.040 (5)	0.043 (7)	0.034 (5)	0.015 (4)	0.005 (3)	−0.005 (5)
C25	0.069 (6)	0.051 (7)	0.043 (5)	0.011 (5)	0.014 (4)	−0.006 (4)
C26	0.084 (8)	0.077 (10)	0.045 (6)	0.020 (7)	0.019 (5)	0.007 (6)
C27	0.075 (8)	0.133 (14)	0.047 (7)	0.030 (8)	−0.004 (6)	−0.013 (7)
C28	0.067 (7)	0.076 (9)	0.092 (9)	−0.002 (6)	−0.002 (6)	−0.033 (7)
C29	0.050 (5)	0.069 (8)	0.054 (6)	0.005 (5)	0.010 (4)	−0.010 (5)
C30	0.089 (8)	0.069 (8)	0.073 (7)	−0.008 (6)	0.028 (6)	−0.009 (6)

### Geometric parameters (Å, °)

**Table:**

I1—C3	2.102 (8)	C11—H11	0.9300
I2—C5	2.095 (8)	C12—C13	1.369 (17)
I3—C18	2.101 (9)	C12—H12	0.9300
I4—C20	2.091 (8)	C13—C14	1.346 (12)
N1—C7	1.259 (9)	C13—H13	0.9300
N1—N2	1.378 (9)	C14—H14	0.9300
N2—C8	1.365 (9)	C15—H15A	0.9600
N2—H2	0.90 (6)	C15—H15B	0.9600
N3—C22	1.284 (9)	C15—H15C	0.9600
N3—N4	1.377 (9)	C16—C21	1.386 (11)
N4—C23	1.341 (10)	C16—C17	1.408 (12)
N4—H4	0.90 (5)	C16—C22	1.456 (11)
O1—C2	1.345 (8)	C17—C18	1.367 (12)
O1—H1	0.8200	C18—C19	1.386 (12)
O2—C8	1.224 (9)	C19—C20	1.367 (12)
O3—C17	1.349 (8)	C19—H19	0.9300
O3—H3	0.8200	C20—C21	1.386 (12)
O4—C23	1.243 (10)	C21—H21	0.9300
C1—C6	1.387 (10)	C22—H22	0.9300
C1—C2	1.420 (11)	C23—C24	1.464 (11)
C1—C7	1.435 (11)	C24—C25	1.381 (12)
C2—C3	1.395 (11)	C24—C29	1.416 (13)
C3—C4	1.383 (10)	C25—C26	1.401 (13)
C4—C5	1.403 (11)	C25—C30	1.507 (13)
C4—H4A	0.9300	C26—C27	1.341 (18)
C5—C6	1.376 (11)	C26—H26	0.9300
C6—H6	0.9300	C27—C28	1.373 (17)
C7—H7	0.9300	C27—H27	0.9300
C8—C9	1.495 (11)	C28—C29	1.409 (12)
C9—C10	1.374 (13)	C28—H28	0.9300
C9—C14	1.412 (12)	C29—H29	0.9300
C10—C11	1.426 (12)	C30—H30A	0.9600
C10—C15	1.450 (13)	C30—H30B	0.9600
C11—C12	1.361 (16)	C30—H30C	0.9600
			
C7—N1—N2	119.1 (7)	C10—C15—H15B	109.5
C8—N2—N1	118.6 (6)	H15A—C15—H15B	109.5
C8—N2—H2	120 (6)	C10—C15—H15C	109.5
N1—N2—H2	121 (6)	H15A—C15—H15C	109.5
C22—N3—N4	118.1 (7)	H15B—C15—H15C	109.5
C23—N4—N3	118.3 (7)	C21—C16—C17	119.7 (8)
C23—N4—H4	113 (6)	C21—C16—C22	119.7 (9)
N3—N4—H4	128 (6)	C17—C16—C22	120.7 (7)
C2—O1—H1	109.5	O3—C17—C18	119.4 (8)
C17—O3—H3	109.5	O3—C17—C16	121.7 (8)
C6—C1—C2	118.3 (8)	C18—C17—C16	118.9 (7)
C6—C1—C7	120.4 (8)	C17—C18—C19	121.3 (9)
C2—C1—C7	121.1 (7)	C17—C18—I3	118.9 (6)
O1—C2—C3	117.8 (7)	C19—C18—I3	119.8 (7)
O1—C2—C1	123.1 (8)	C20—C19—C18	119.8 (9)
C3—C2—C1	119.1 (7)	C20—C19—H19	120.1
C4—C3—C2	121.8 (7)	C18—C19—H19	120.1
C4—C3—I1	119.7 (6)	C19—C20—C21	120.3 (8)
C2—C3—I1	118.4 (6)	C19—C20—I4	120.8 (7)
C3—C4—C5	118.6 (8)	C21—C20—I4	118.9 (7)
C3—C4—H4A	120.7	C16—C21—C20	120.0 (9)
C5—C4—H4A	120.7	C16—C21—H21	120.0
C6—C5—C4	120.1 (7)	C20—C21—H21	120.0
C6—C5—I2	121.2 (6)	N3—C22—C16	120.6 (9)
C4—C5—I2	118.6 (6)	N3—C22—H22	119.7
C5—C6—C1	121.9 (8)	C16—C22—H22	119.7
C5—C6—H6	119.0	O4—C23—N4	121.0 (9)
C1—C6—H6	119.0	O4—C23—C24	122.9 (7)
N1—C7—C1	119.2 (8)	N4—C23—C24	115.9 (7)
N1—C7—H7	120.4	C25—C24—C29	119.7 (8)
C1—C7—H7	120.4	C25—C24—C23	122.8 (9)
O2—C8—N2	121.4 (8)	C29—C24—C23	117.5 (8)
O2—C8—C9	124.7 (7)	C24—C25—C26	119.1 (10)
N2—C8—C9	113.9 (6)	C24—C25—C30	121.3 (8)
C10—C9—C14	121.1 (8)	C26—C25—C30	119.5 (9)
C10—C9—C8	120.3 (8)	C27—C26—C25	120.7 (11)
C14—C9—C8	118.5 (9)	C27—C26—H26	119.7
C9—C10—C11	117.2 (9)	C25—C26—H26	119.7
C9—C10—C15	125.3 (8)	C26—C27—C28	122.7 (11)
C11—C10—C15	117.4 (9)	C26—C27—H27	118.6
C12—C11—C10	120.1 (10)	C28—C27—H27	118.6
C12—C11—H11	120.0	C27—C28—C29	118.1 (11)
C10—C11—H11	120.0	C27—C28—H28	120.9
C11—C12—C13	121.6 (11)	C29—C28—H28	120.9
C11—C12—H12	119.2	C28—C29—C24	119.7 (9)
C13—C12—H12	119.2	C28—C29—H29	120.2
C14—C13—C12	120.0 (11)	C24—C29—H29	120.2
C14—C13—H13	120.0	C25—C30—H30A	109.5
C12—C13—H13	120.0	C25—C30—H30B	109.5
C13—C14—C9	120.0 (10)	H30A—C30—H30B	109.5
C13—C14—H14	120.0	C25—C30—H30C	109.5
C9—C14—H14	120.0	H30A—C30—H30C	109.5
C10—C15—H15A	109.5	H30B—C30—H30C	109.5

### Hydrogen-bond geometry (Å, °)

**Table:**

*D*—H···*A*	*D*—H	H···*A*	*D*···*A*	*D*—H···*A*
O1—H1···N1	0.82	1.87	2.570 (9)	143
O3—H3···N3	0.82	1.85	2.560 (10)	144
N4—H4···O2	0.90 (5)	1.94 (4)	2.786 (8)	155 (8)
N2—H2···O4^i^	0.90 (6)	1.91 (3)	2.788 (9)	164 (10)

Symmetry codes: (i) *x*−1, *y*, *z*.
